# Characterisation of feline renal cortical fibroblast cultures and their transcriptional response to transforming growth factor β1

**DOI:** 10.1186/s12917-018-1387-2

**Published:** 2018-03-09

**Authors:** J. S. Lawson, H. M. Syme, C. P. D. Wheeler-Jones, J. Elliott

**Affiliations:** 1 Comparative Biomedical Sciences, The Royal Veterinary College, Royal College Street, London, NW1 0TU UK; 2 Clinical Sciences and Services, The Royal Veterinary College, Hawkshead Lane, North Mymms, Hatfield, Herts AL9 7TA UK

**Keywords:** CKD, Cats, Renal fibrosis, Myofibroblast

## Abstract

**Background:**

Chronic kidney disease (CKD) is common in geriatric cats, and the most prevalent pathology is chronic tubulointerstitial inflammation and fibrosis. The cell type predominantly responsible for the production of extra-cellular matrix in renal fibrosis is the myofibroblast, and fibroblast to myofibroblast differentiation is probably a crucial event. The cytokine TGF-β1 is reportedly the most important regulator of myofibroblastic differentiation in other species. The aim of this study was to isolate and characterise renal fibroblasts from cadaverous kidney tissue of cats with and without CKD, and to investigate the transcriptional response to TGF-β1.

**Results:**

Cortical fibroblast cultures were successfully established from the kidney tissue of cats with normal kidney function (FCF) and cats with chronic kidney disease (CKD-FCF). Both cell types expressed the mesenchymal markers vimentin, CD44 and CD29, and were negative for the epithelial marker cytokeratin, mesangial cell marker desmin and endothelial cell marker vWF. Only CKD-FCF expressed VCAM-1, a cell marker associated with inflammation. Incubation with TGF-β1 (0–10 ng/ml) induced a concentration dependent change in cell morphology, and upregulation of myofibroblast marker gene α-SMA expression alongside collagen 1α1, fibronectin, TGF-β1 and CTGF mRNA. These changes were blocked by the TGF-β1 receptor 1 antagonist SB431542 (5 μM).

**Conclusions:**

FCF and CKD-FCF can be cultured via a simple method and represent a model for the investigation of the progression of fibrosis in feline CKD. The findings of this study suggest TGF-β1 may be involved in fibroblast-myofibroblast transition in feline CKD, as in other species.

## Background

Chronic kidney disease (CKD) is common in geriatric cats, with a reported prevalence of 28–50% [1, 2]. The majority of cats with CKD are found to have non-specific renal lesions and the predominant morphological diagnosis in these cases is chronic tubulointerstitial inflammation and fibrosis [3, 4]. Whilst fibrosis is a normal sequelae of injury, it is thought that in CKD the normal wound healing response fails to terminate [5, 6] and the expansion of the extra-cellular matrix (ECM) gradually destroys normal tissue structure [7]. The composition of the aberrant ECM in cats with CKD has not been well defined, but both collagen I and fibronectin have been shown to be upregulated in the diseased renal interstitium [8, 9].

In the normal kidney, the dominant cell type responsible for ECM homeostasis is the interstitial fibroblast [10]. Interstitial fibroblasts play an important role in the synthesis and degradation of extra-cellular matrix as well as an endocrine role as producers of erythropoietin [11] and, under certain conditions, renin [12]. Additionally, fibroblasts play an important role in maintaining the integrity of the renal vasculature. In renal fibrosis the major ECM producing cell is the myofibroblast, a cell type which possesses both fibroblastic and smooth muscle cell characteristics [13, 14]. Although the origin of these cells has previously been controversial, they are now believed to originate primarily from resident fibroblasts [14]. The morphological features differentiating a myofibroblast from a fibroblast are only apparent on electron microscopy and expression of the contractile protein α-SMA is the most widely used marker of myofibroblast differentiation. Interstitial α-SMA expression in cats is reported to appear early in the pathogenesis of CKD and is correlated with severity of fibrosis and serum creatinine [8, 15].

The induction and proliferation of myofibroblasts is probably a crucial event in the initiation and progression of renal fibrosis, and may be regulated by a number of local and circulating factors. These include paracrine fibroblast growth factors (FGFs), platelet-derived growth factor (PDGF), angiotensin II, aldosterone and, most importantly, transforming growth factor β1 (TGF-β1) and connective tissue growth factor (CTGF) [16]. TGF-β1 is a pro-fibrotic cytokine thought to be the primary mediator driving the progression of interstitial fibrosis in chronic kidney disease, which has led to its description as the “master regulator of fibrosis” [17]. TGF-β1 directly stimulates transcription of ECM, as well as mediating effects via downstream activation of the matricellular protein connective tissue growth factor (CTGF) [18, 19]. Increased urinary TGF-β1 excretion is associated with interstitial fibrosis in cats [20], suggesting this cytokine may play a causative role in the development of fibrosis as in other species. However, whether TGF-β1 has analogous effects on feline renal fibroblasts at a cellular level is unknown.

Cultures of renal fibroblasts are recognised as an important tool for understanding the fibroblast-myofibroblast transition and regulation of ECM production in renal fibrosis [21]. However, there are currently no published studies detailing a methodology for the culture of feline fibroblasts, or examining the effects of pro-fibrotic mediators such as TGF-β1 on these cells. The aims of this study were to:

i) Isolate and characterise renal cortical fibroblasts from the cadaverous renal tissue of cats with normal renal function and cats with CKD
ii) Determine the effect of TGF-β1 on mRNA expression of genes associated with activation and differentiation towards myofibroblast phenotype in cortical fibroblasts isolated from cats with chronic kidney disease (CKD-FCF)

## Methods

### Cell culture

Kidneys were obtained from three cats with CKD and two cats with normal renal function recently (< 2 h) euthanased for welfare reasons, with owner informed consent. Cats were diagnosed with CKD based upon history, physical examination and a plasma creatinine concentration > 177 μmol/L with concurrent urine specific gravity < 1.035. Cats were considered to have normal kidney function in the absence of any historical or physical examination findings suggest of kidney disease, and if plasma creatinine was < 177 μmol/L with appropriately concentrated urine.

The renal cortex was minced and dissociated by incubating in Dulbecco’s modified Eagle medium: Nutrient mixture F-12 (DMEM/F12) containing 1 mg/ml collagenase (collagenase A from *clostridium hemolyticum*, Roche) for 25 min at 37 °C in a water bath. The digested tissue was centrifuged for 3 min at 90 x g, resuspended in DMEM/F12 and passed through a 212-μm sieve set on top of a 106-μm sieve into a 100 mm tissue culture dish. The sieved cells were centrifuged for 3 min at 90 x *g* and resuspended in 10 ml DMEM/F12. The resuspended mixture was left to settle for 10 min, and the supernatant removed in order to remove residual red blood cells. The remaining pellet was resuspended in DMEM/F12 containing 10% foetal bovine serum (FBS) and 1% penicillin-streptomycin (Thermofisher scientific) and transferred into a 75cm^2^ tissue culture flask (Nunclon™ delta surface). Cells were maintained at 37 °C in a 5% CO_2_/95% air humidified incubator (BB15 CO_2_; Thermofisher scientific). After 24 h the medium was removed and the cells gently washed with Dulbecco’s phosphate buffered saline (DPBS; Life technologies) before replacement of fresh primary culture medium. Thereafter, culture medium was replaced every 48 h. Once 75–80% confluent, the cells were trypsinized and distributed into tissue culture flasks/plates at a 1:3 subculture ratio. For experimental use, cells were cultured in reduced serum medium containing 3% FBS. All experiments were performed using cells from passages 1 or 2.

### Immunofluorescent detection of proteins in isolated cells

Cells were assessed for the expression of the marker proteins cytokeratin AE1/AE3, vimentin, desmin, von Willebrand factor, CD44, CD29 and VCAM-1 by immunofluorescence. Cells were plated onto collagen I coated glass bottomed chamber slides (Nunc Lab-tek II chamber slide™, Sigma-Aldrich), left to adhere, then fixed in 4% formaldehyde for 15 min. Fixed cells were incubated with 50 mM ammonium chloride (Sigma-Aldrich) for 15 min, washed three times with tris-buffered saline (TBS) and permeabilized with 0.1% triton in TBS. Cells were incubated in blocking buffer (3% BSA, 1% goat serum, 0.1% tween in TBS) for 2 h at room temperature, then incubated with the desired primary antibody diluted in blocking reagent for 1 h at room temperature. Details of the primary antibodies used are provided in Table 1. After three washes with TBS, labelled cells were incubated with the respective fluorescent secondary antibody (1:1000 in blocking reagent) for 1 h in the dark. After three further washes with TBS pH 7.4, the chambers were removed and a coverslip mounted using Fluoroshield™ with DAPI (Sigma-Aldrich). Images were collected using a DM4000B upright microscope with samples illuminated using an EBQ100 light source and filter cubes A4, L5 and TX2 (all from Leica Microsystems, Milton Keynes, UK) and an AxioCam MRm monochrome camera controlled through Axiovision software version 4.8.2 (Carl Zeiss Ltd., Cambridge, UK). Each experimental repeat included an isotype control matched to the species and isotype of the primary antibody used. Experiments were carried out in triplicate for the CKD-FCF and duplicate for the FCF.

**Table 1:** Primary antibodies used for immunofluorescence and western blotting

Antibody	Supplier	Secondary antibody	Dilution for IF	Dilution for western blot	Incubation temperature for western blot
Cytokeratin AE1/AE3	Dako	Mouse	1:100	1:1000	4 °C
Vimentin (V9)	Dako	Mouse	1:100	1:1000	4 °C
Desmin (DE-R-11)	Dako	Mouse	1:100	N/A	N/A
CD29 (TS2/16)	Bio-rad	Mouse	1:100	1:500	4 °C
CD44 (156-3C11)	ABD serotec	Rat	1:100	N/A	N/A
vWF	Dako	Rabbit	1:200	1:1000	4 °C
VCAM-1	R&D	Mouse	1:100	N/A	N/A
Rabbit Polyclonal Isotype control	Biolegend	Rabbit	1:50–1:200	N/A	N/A
Mouse IgG1 κ isotype control	Biolegend	Mouse	1:50–1:200	N/A	N/A
β-actin (AC-74)	Sigma-Aldrich	Mouse	N/A	1:5000	4 °C

### Western blotting

Confluent monolayers of CKD-FCF were washed with ice cold DPBS, containing 0.4 mM sodium orthovanadate (Sigma-Aldrich), before lysis in 68.3 mM Tris-HCl (Sigma-Aldrich) containing 10% (*w*/*v*) glycerol (Sigma-Aldrich), 2% (w/v) sodium dodecyl sulphate (SDS; Sigma-Aldrich), 2 mM sodium orthovanadate, 10 μl/ml protease inhibitor cocktail (Sigma-Aldrich). Proteins (25 μg) were separated by sodium dodecyl sulfate polyacrylamide gel electrophoresis (SDS-PAGE) using pre-cast gels (10% Mini-protean TGX Stain-Free™ protein gel, Bio-rad), before transfer onto a polyvinylidene difluoride (PVDF) membrane (0.45 μm pore, Hybond™; GE Healthcare). Membranes were blocked for 3 h at room temperature in 5% (*w*/*v*) milk (Marvel) made up in Tris-buffered saline with Tween (TBST; 50 mM Tris, 150 mM NaCl, 0.02% (*v*/v) Tween 20, pH 7.4). For immunodetection of proteins of interest, membranes were incubated overnight at 4 °C with primary antibody diluted in TBST + 10% (*w*/*v*) BSA containing anti-vimentin (1:1000), anti-cytokeratin AE1/AE3 (1:1000), anti-vWF (1:1000), anti-CD29 (1:500), or anti-β-actin (1:5000) antibody. Subsequent to this, membranes were washed in TBST (6 × 10 min) and then incubated with horse radish peroxidase (HRP) conjugated goat anti-rabbit/mouse IgG as appropriate (1:10,000) in TBST containing 0.2% (*w*/*v*) BSA for 1 h at room temperature. After further washing (6 × 10 min), immunoreactive bands were detected via enhanced chemiluminescence (ECL). This was performed by exposing the blots to 0.1 M Tris pH 8.5 containing 0.25 mg/ml luminol (Sigma-Aldrich), 0.18 mg/ml 4-iodophenol (Sigma-Aldrich), 0.01% (*v*/v) H_2_O_2_ (Sigma-Aldrich) for 1 min, and visualising bands using photographic film (Hyperfilm™, Kodak). Experiments were carried out in triplicate, where each experimental repeat represented a separate isolation from an individual cat.

### Evaluation of cell morphology after incubation with TGF-β1

CKD-FCF were incubated for 72 h in six-well tissue culture plates (Nunclon™ delta surface) in the presence of vehicle, 0.1–10 ng/ml human recombinant TGF-β1 (rTGF-β1) or 10 ng/ml rTGF-β1 plus the TGF-β1 receptor 1 (TGF-β1R1) antagonist SB431542 (5 μM). Images of the incubated cells were collected using a DMIRB inverted microscope with samples illuminated using an EBQ100 light source (Leica Microsystems, Milton Keynes, UK) and an AxioCam ICm1 monochrome camera controlled through Axiovision software version 4.8.2 (Carl Zeiss Ltd., Cambridge, UK). Longitudinal and transverse cell diameter was assessed by measuring 25 cells in each image using commercially available software (ImageJ) and obtaining the mean. Experiments were performed in triplicate, where each experimental repeat represented a separate isolation from an individual cat. Statistical significance was evaluated by one-way analysis of variance (ANOVA) with *post-hoc* Dunnet’s test. Statistical analyses were performed using GraphPad Prism software version 6.0 (Graphpad Software, La Jolla, CA).

### RT-qPCR analysis of gene expression after incubation with TGF-β1

RNA from cells treated as previously described was extracted using a column based kit (Genelute™ Mammalian Total RNA Miniprep Kit, Sigma-Aldrich). Messenger RNA (mRNA) templates were reverse transcribed to complementary DNA (cDNA) using a commercially available kit (Omniscript RT, Qiagen), oligo dT primer (MWG Eurofins) and 1 U/ml RNaseOUT (Life Tehnologies). Gene expression was quantified by RT-qPCR in 96-well plates (Framestar™, Fortitude) using a commercially available SYBR Green *Taq* ready mix (SYBR® Green JumpStart™ Taq ReadyMix™, Sigma-Aldrich), and was performed in a CFX Connect™ Real-Time PCR Detection System (Bio-Rad).

Collagen type 1α1 (Col1α1), Fibronectin, α-SMA, CTGF and TGF-β1 gene expression was analysed by RT-qPCR and normalised to GAPDH/RPS7 using primers listed in Table 2. Experiments were performed in quadruplicate, where each experimental repeat represented a separate isolation from an individual cat. Data are expressed as mean fold change relative to untreated control and statistical significance was evaluated by one-way analysis of variance (ANOVA) with *post-hoc* Dunnet’s test. Statistical analyses were performed using GraphPad Prism software version 6.0 (Graphpad Software, La Jolla, CA).

**Table 2:** Primer sequences

Gene	Primer sequence (forward primer first, 5′ to 3′)	Amplicon size (bp)	GC Content (%)	Annealing temp	Primer dilution
*RPS7* ^*a*^	GTCCCAGAAGCCGCACTTTGAC CTCTTGCCCACAATCTCGCTCG	82	59 59	60.8	1:10
*GAPDH* ^*a*^	AGTATGATTCCACCCACGGCA GATCTCGCTCCTGGAAGATGGT	101	5255	64.1	1:10
*COL1A1*	GAGAGCATGACCGACGGATT TAGGTGATGTTCTGGGACGC	122	55 55	58.7	1:10
*TGFB1* ^*b*^	GGAATGGCTGTCCTTTGATG TGCAGTGTGTTATCTTTGCTGTC	120	50 43.5	60	1:10
*CTGF*	GGAAGACACATTTGGCCCAG GCTTCTCCAACCTGCAGAAG	146	55 55	62.1	1:10
*FN1*	CCCTCACCAATCTCACTCCA CCCTCGGAACATCAGAAACTG	117	55 52.4	58	1:10

^a^Primer obtained from previous publication: Penning et al. [56]

^b^Primer obtained from previous publication: Nguyen Van et al. [57]

## Results

### Characterisation of feline cortical fibroblast (FCF) cultures

**Fig. 1:**
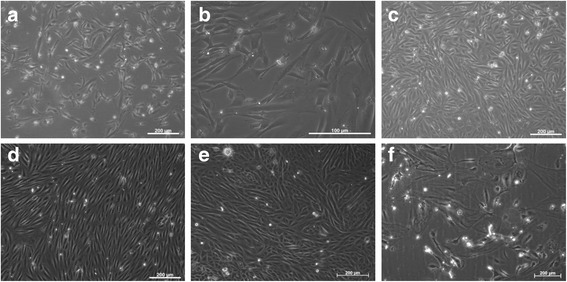
Morphology of cortical fibroblasts isolated from cats with normal kidney function (FCF) and CKD (CKD-FCF). Representative photomicrographs of FCF. **a**, **b**: CKD-FCF 72 h post isolation. In the presence of FBS, randomly orientated fusiform cells proliferate and begin to dominate the culture **c**: CKD-FCF 7 days post isolation. Prior to the first passage, although the predominating cell type appears fibroblastic, there are still isolated pockets of epithelial cells. **d**: CKD-FCF, passage 1: Relatively homogenous monolayers of cells in parallel arrays are present in cultures derived from diseased kidneys **e**: FCF, passage 1: In cultures derived from cats with normal kidney function, cultures at passage 1 are still heterogenous and include pockets of epithelial cells. These cultures are homogenous in nature by passage 2. **f**: CKD-FCF, passage 5: After 4–9 passages, cells developed replicative senescence and underwent a change in morphology. Images were collected using a DMIRB inverted microscope with samples illuminated using an EBQ100 light source (Leica Microsystems, Milton Keynes, UK) and an AxioCam ICm1 monochrome camera controlled through Axiovision software version 4.8.2 (Carl Zeiss Ltd., Cambridge, UK)

Cultures were successfully established from the kidney tissue of two cats with normal kidney function (FCF) and four cats with chronic kidney disease (CKD-FCF). Initial primary cultures were heterogeneous; however where the tissue was taken from cats with CKD these cultures were rapidly dominated by cells of a slender fusiform morphology within 7 days and after the first passage the culture was relatively homogenous (Fig. 1a-d). Cultures from the tissue of cats with normal kidney function were initially dominated by cells of a cobblestone morphology, and it took until passage 2 to obtain homogenous cultures of fusiform cells (Fig. 1e). At lower cell densities, cells exhibited multi-polar and bi-polar morphology and were randomly orientated but, at confluence, all cells exhibited a bi-polar morphology and formed parallel arrays and whorls. Cells maintained their morphology, and continued to proliferate until passage 4–9 when they became enlarged and senescent (Fig. 1f).

**Fig. 2:**
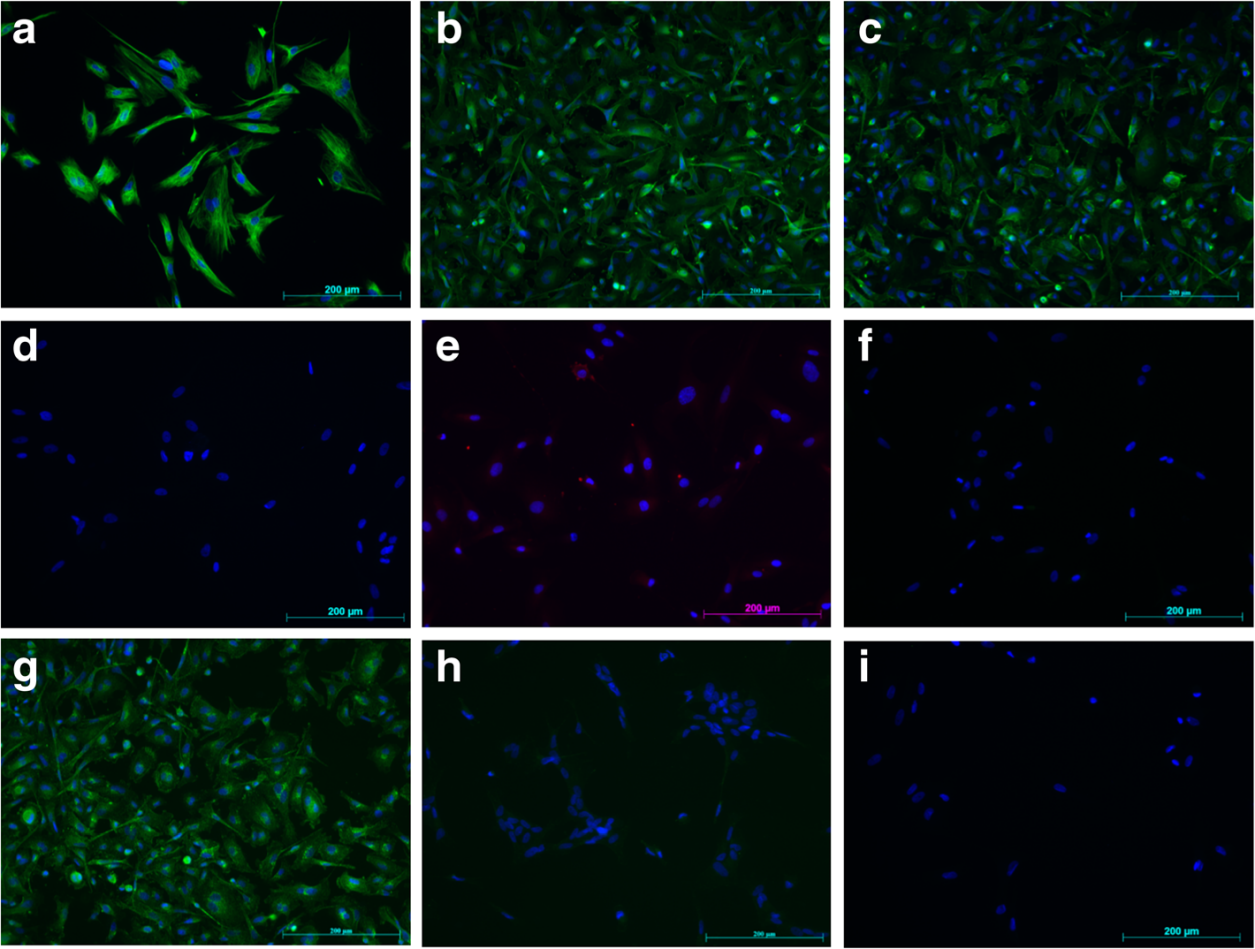
Immunofluorescence studies of feline cortical fibroblasts isolated from healthy (FCF) and diseased kidneys (CKD-FCF). Immunofluorescence staining of FCF and CKD-FCF. Cell nuclei were stained with DAPI (blue). Both CKD-FCF and FCF stained positive for the mesenchymal markers vimentin (**a**), CD29 (**b**) and CD44 (**c**), and were negative for the epithelial marker cytokeratin AE1/AE3 (**d**), endothelial cell marker vWF (**e**), and the myogenic marker desmin (**f**) (images from CKD-FCF shown). CKD-FCF demonstrated VCAM-1 expression (**g**) in addition to this, but FCF isolated from the kidneys of healthy cats did not (**h**). Isotype controls were negative (**i**). Images were collected using a DM4000B upright microscope with samples illuminated using an EBQ100 light source and filter cubes A4 and L5 (all from Leica Microsystems) and an AxioCam MRm monochrome camera controlled through Axiovision software version 4.8.2 (Carl Zeiss Ltd). Images are representative of cells isolated from 3 different cats (CKD-FCF) and 2 different cats (FCF)

**Fig. 3:**
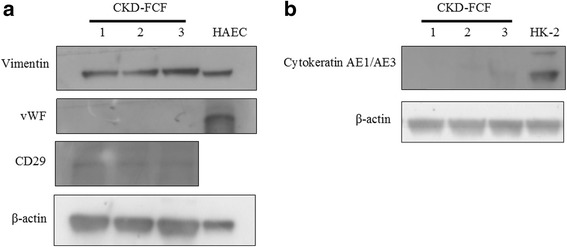
Western blots of CKD-FCF lysates. Immunoblots of CKD-FCF protein lysates from 3 separate isolations. **a**. CKD-FCF show strong expression of the mesenchymal marker vimentin and weaker expression of the mesenchymal marker CD29. There was no expression of the endothelial marker vWF. Human endothelial cell lysate (HAEC) was used as a positive control. **b**. CKD-FCF do not express the epithelial marker cytokeratin AE1/AE3. Lysates of human tubular epithelial cells (HK-2) were used as a positive control

Cells were further characterised using immunofluorescence, with western blots performed on CKD-FCF lysates to confirm specificity of antibodies used. FCF and CKD/FCF stained uniformly positive for vimentin on immunocytochemistry (Fig. 2a). The vimentin antibody also recognised a band of approximate molecular weight 54 kDa in CKD-FCF lysates (Fig. 3a). Both FCF and CKD-FCF were positive for CD29 by immunofluorescence (Fig. 2b). The CD29 antibody recognised a band of approximate molecular weight 130 kDa via immunoblotting (Fig. 3a). FCF/CKD-FCF were positive for CD44 (Fig. 2c), CD44 could not be detected by immunoblotting (data not shown). FCF/CKD-FCF were uniformly negative for cytokeratin AE1/AE3 by immunofluorescence (Fig. 2d), and no cytokeratin protein could be detected in CKD-FCF protein lysates by immunoblotting (Fig. 3b). A human tubular epithelial cell line (HK-2) was obtained (CRL-2190, ATCC) and cultured as per the manufacturer’s protocol for use as a positive control [22].Both cell types were negative for desmin expression by immunocytochemistry (Fig. 2f). Desmin could not be detected via immunoblotting in CKD-FCF lysates (data not shown). FCF/CKD-FCF were negative for vWF expression by immunofluorescence (Fig. 2e), and immunoblotting could not detect vWF in CKD-FCF (Fig. 3a). Human umbilical vein endothelial cells (HUVEC) were used as a positive control. Human umbilical cord collection (obtained with informed written consent) conformed to the principles outlined in the Declaration of Helsinki and is approved by the NHS Health Research Authority East of England-Cambridge South Research Ethics Committee (REC reference 16/EE/0396). HUVEC were isolated and cultured as described previously and were used at passage 2 [23]. CKD-FCF were positive (Fig. 2g) whilst FCF were negative for VCAM-1 on immunofluorescence (Fig. 2h). VCAM-1 could not be detected by immunoblotting in cell culture lysates or a positive control consisting of activated endothelial cells (data not shown). A summary of marker expression for both cell types can be found in Table 3.

**Table 3:** Summary of marker expression in isolated feline cells and cell lines

Marker	FCF	CKD-FCF
Cytokeratin (AE1/AE3)	–	–
Vimentin	+	+
Desmin	–	–
vWF	–	–
CD29	+	+
CD44	+	+
VCAM-1	–	+

### Response of CKD-FCF to incubation with TGF-β1

**Fig. 4:**
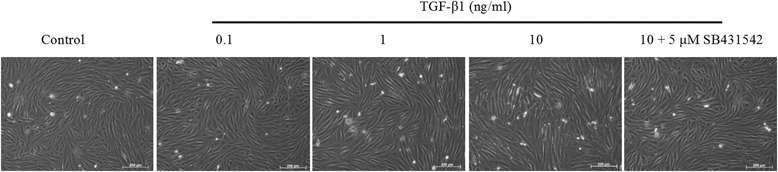
Representative example of the morphological changes in CKD-FCF after 72 h incubation with TGF-β1. Representative photomicrographs of CKD-FCF cells after incubation with 0–10 ng/ml TGF-β1 for 72 h. The CKD-FCF underwent dose-dependent morphological changes, becoming hypertrophic and elongatedThese alterations were inhibited by 5 μM of the TGF-β1R1 antagonist SB431542. Images were collected using a DMIRB inverted microscope with samples illuminated using an EBQ100 light source (Leica Microsystems) and an AxioCam ICm1 monochrome camera controlled through Axiovision software version 4.8.2 (Carl Zeiss Ltd)

**Fig. 5:**
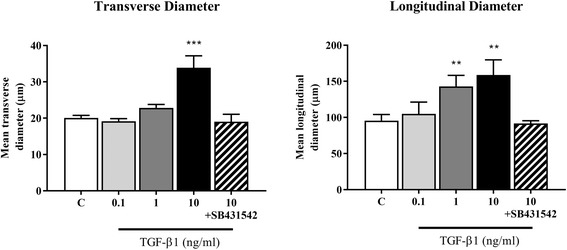
Transverse and Longitudinal diameter of CKD-FCF after 72 h incubation with TGF-β1. Transverse and longitudinal diameter of CKD-FCF cells was assessed after incubation with TGF-β1 (0.1 to 10 ng/ml). There was a significant increase in mean cell longitudinal diameter after incubation with 1 ng/ml TGF-β1, and increases in both mean cell longitudinal and transverse diameter after incubation with 10 ng/ml TGF-β1. These alterations were inhibited by 5 μM of the TGF-β1R1 antagonist SB431542. Data represent three experimental repeats using cells from different cats and were analysed using the one-way ANOVA with Dunnett’s post-hoc analysis. The columns represent mean length and error bars represent the standard deviation. **P < 0.01 ***P < 0.001

Experiments to assess the morphological and transcriptional response to TGF-β1 were performed on CKD-FCF only, as there were insufficient FCF isolations available to obtain statistical significance. TGF-β1 incubation resulted in alterations in CKD-FCF cell morphology, with a concentration dependent hypertrophic effect evident (Fig. 4). Incubation with 1 ng/ml TGF-β1 for 72 h resulted in an increase in mean cell longitudinal diameter, and incubation with 10 ng/ml TGF-β1 resulted in increases in both mean cell longitudinal and transverse diameter (Fig. 5). All morphological changes were abrogated by the addition of 5 μM SB431542.

**Fig. 6:**
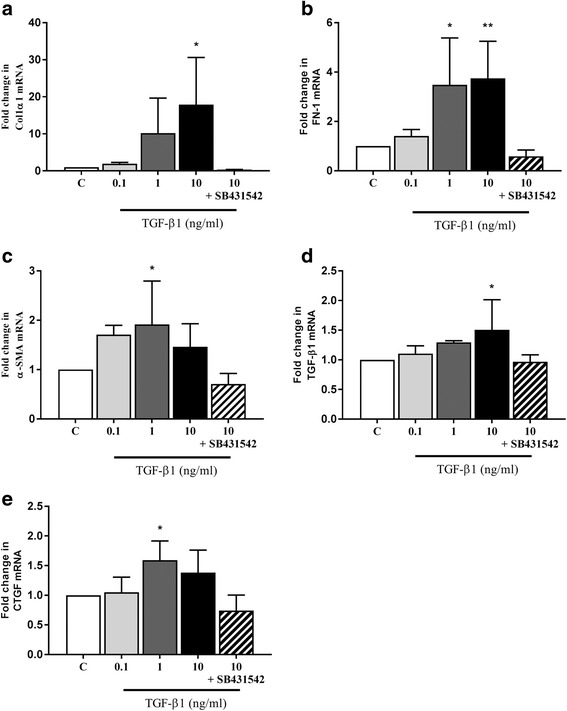
TGF-β1 mediated expression of genes related to myofibroblast induction, ECM production and downstream signalling in CKD-FCF. Expression of the myofibroblast marker α-SMA, ECM-related genes and downstream mediator mRNA was assessed in the CKD-FCF by RT-qPCR after incubation with TGF-β1 (0.1 to 10 ng/ml). Target gene mRNA copy number was normalised to GAPDH/RPS7 and is expressed as fold change in relation to control. There were significant increases in collagen type 1α1 expression (**a**), fibronectin expression (**b**), α-SMA expression (**c**), TGF-β1 expression (**d**) and CTGF expression (**e**). There were no significant changes in gene expression when cells were incubated with 10 ng/ml TGF-β1 in the presence of 5 μM SB431542. Data represent four experimental repeats using cells from different cats and were analysed using the one-way ANOVA with Dunnett’s *post-hoc* analysis. The columns represent the mean normalised mRNA copy number and error bars represent the standard deviation.*P < 0.05 **P < 0.01

There were no significant changes in target gene expression after incubation with 0.1 ng/ml TGF-β1 for 72 h. Incubation with 1 ng/ml of TGF-β1 for 72 h resulted in significant increases in α-SMA (*P* = 0.044), fibronectin (*P* = 0.0199), and CTGF expression (*P* = 0.0272). The higher concentration of 10 ng/ml TGF-β1 induced increased expression of fibronectin (*P* = 0.01) collagen type 1α1 (*P* = 0.0145) and TGF-β1 (*P* = 0.0109) after 72 h (Fig. 6a-e). In the presence of 5 μM SB431542, alterations in gene expression induced by 10 ng/ml of TGF-β1 were completely inhibited.

## Discussion

This study describes a simple protocol for the successful culture of cortical fibroblasts from the kidneys of cats with normal kidney function, and cats with chronic kidney disease. To the author’s knowledge, this is the first study to describe feline renal fibroblasts in culture, although previously methodology has been published for the isolation and characterisation of these cells from a number of other species, including the rabbit [24], rat [25], mouse [26], and human [27]. It has been recognised since the earliest days of tissue culture that, in the absence of defined serum-free medium, fibroblasts will proliferate and overgrow cultures of epithelial cells [28]. This phenomenon was used to obtain cultures of feline fibroblasts by seeding the sieved cortical tubulointerstitial cell fraction into medium containing 10% foetal bovine serum, a method used previously to obtain fibroblasts from human kidneys [27]. Using tissue from cats with CKD, these cultures were almost completely dominated by cells of a fibroblastic morphology by passage one, whereas in cats with normal kidney function this did not occur until passage two. This discrepancy is probably due to the number of resident fibroblasts present in the initial tissue; in normal cats the interstitial expression of fibroblast/myofibroblast markers is low, but expression increases in cats with CKD [8, 15].

The renal fibroblast population in situ is recognised to be heterogeneous, with a number of diverse functions, and is historically not well defined [29, 30]. Collagen expressing cells surrounding arteries and venules are known as perivascular fibroblasts, and those closely associated with peritubular capillaries via pedicle like attachment plaques are known as pericytes [31]. There are no markers which exclusively and specifically label all fibroblasts [29, 32], and differentiating between subpopulations remains challenging. There is confusion in the field regarding the precise identity and function of renal fibroblasts, and in the present study a pragmatic definition of renal fibroblasts was taken, based on a recent review, as “nonvascular, nonepithelial, and noninflammatory cell constituents of the kidney tubulointerstitium” [30]. The isolated FCF/CKD-FCF in the present study demonstrated an expression profile similar to that previously reported for renal fibroblasts from other species, namely positive for the mesenchymal markers vimentin, CD44 (a transmembrane adhesion glycoprotein) and CD29 (β_1_ integrin), and negative for the epithelial marker cytokeratin, mesangial cell marker desmin and endothelial cell marker vWF [27, 33, 34]. Vimentin is the major intermediate filament protein of mesenchymal cells [35], and has previously been used as a fibroblast marker in the normal feline kidney [15]. The transmembrane proteins CD44 and CD29 have also previously been shown to label almost 100% of feline dermal fibroblasts [36]. The CKD-FCF expressed vascular cell adhesion molecule 1 (VCAM-1), whereas FCF were negative for this marker. VCAM-1 is an adhesion molecule of the immunoglobulin superfamily normally expressed on monocytes and endothelial cells, which can be upregulated in fibroblasts exposed to pro-inflammatory cytokines such as TNF-α [37]. The results in the present study are in accordance with previous work, where renal fibroblasts in healthy kidneys were negative for VCAM-1 but those in kidneys associated with inflammatory infiltrates or fibrosis upregulated expression of this adhesion molecule [33, 38]. Therefore, it is possible that the CKD-FCF in the present study represent fibroblasts in an “activated” state. Further characterisation of these cells could include examining expression of marker proteins such as α-SMA, to determine whether they represent a population of myofibroblasts, however this may be unlikely as myofibroblasts have been reported to have a low proliferative capacity in vitro [39].

Resident renal fibroblasts are the cell type predominantly responsible for the production of extra-cellular matrix in renal fibrosis, through differentiation into myofibroblasts [14]. This differentiation is primarily thought to occur subsequent to signalling from injured tubular epithelial cells, as well as infiltrating mononuclear cells, via a variety of paracrine cytokines and growth factors [40, 41]. The morphological features differentiating a myofibroblast from a fibroblast are only apparent on electron microscopy and the contractile protein α-SMA is the most widely used marker of myofibroblast differentiation. This protein is associated with the role of the myofibroblast in tissue contraction and interstitial α-SMA expression has been demonstrated to be upregulated in the renal interstitium of cats with CKD [15]. In the present study, mRNA expression of α-SMA in CKD-FCF was significantly upregulated by incubation with 1 ng/ml TGF-β1, suggesting an activation of these cells towards the myofibroblast phenotype. Treated cells also exhibited a concentration-dependent cellular hypertrophy in response to TGF-β1, which has been noted in previous studies and may also be associated with an activated phenotype [42]. This result is in agreement with previous in vitro studies utilising fibroblasts from other species, where TGF-β1 is well characterised as a mediator of fibroblast to myofibroblast transition [43].

The upregulation of α-SMA mRNA expression in the fibroblasts was accompanied by increased collagen type 1α1 and fibronectin mRNA expression, which is a feature consistent with the myofibroblast phenotype. Collagen type I is the main structural element of the normal cortical interstitial matrix, and has been demonstrated to accumulate in increased quantities in the interstitium of cats with CKD, where it is the predominant collagen isoform [44, 45]. TGF-β1 has been shown to directly upregulate collagen transcription in fibroblasts isolated from other species [46, 47], and it would appear that TGF-β1 can also be considered a potent inducer of collagen type I transcription in feline renal fibroblasts. Fibronectin is a large, adhesive glycoprotein, which appears early on in the fibrotic process where it is thought to form a scaffold for the deposition of other ECM proteins and acts as a fibroblast chemoattractant [7]. Fibronectin has previously been reported to be present in increased quantities in the renal interstitium of cats with chronic kidney disease [8]. The source of fibronectin in experimental models of renal disease has been shown to be interstitial myofibroblasts [48], and fibronectin has also been correlated with α-SMA expression in cats [8]. In vitro, TGF-β1 has previously been reported to induce fibronectin production from fibroblasts derived from other species [47], and the results of the present study suggest TGF-β1 may be involved in the production of fibronectin by myofibroblasts in feline kidney disease also.

Treatment with recombinant TGF-β1 protein resulted in auto-induction of TGF-β1 mRNA. This phenomenon has been documented previously, and is thought to be important in amplifying and sustaining the effects of TGF-β1 via an autocrine loop [49]. Incubation with 1 ng/ml TGF-β1 also resulted in increased in CTGF mRNA expression. CTGF is a member of the CCN family of matricellular proteins, a group which plays an important role in tissue homeostasis, wound healing and repair via mediation of ECM-cell crosstalk and modulation of growth factor activity [50]. CTGF is thought to be an important downstream mediator of the pro-fibrotic effects of TGF-β1, particularly myofibroblast activation and ECM production [50, 51]. The current study suggests CTGF may also be involved in TGF-β1 mediated fibrogenesis in the feline kidney as in other species, although further work is needed to demonstrate a causative role.

The canonical TGF-β1 signalling pathway involves TGF-β1 binding to the TGF-β1 type II receptor (TGF-β1RII), which recruits the TGF-β1 type I receptor (TGF-β1RI, also known as activin-like kinase 5 [ALK-5]) and forms a heteromeric receptor complex which phosphorylates receptor regulated Smad proteins (Smad2 and Smad3) [52]. These proteins then go on to regulate the transcription of target genes in the nucleus. TGF-β1-induced collagen production has been reported to be dependent on TGF-β1RII in renal interstitial matrix-producing cells, both in vitro and in vivo [53]. In the present study, the small molecule TGF-β1RI antagonist SB431542 was found to inhibit the pro-fibrotic effects of TGF-β1 on CKD-FCF at the mRNA level. This finding is in agreement with previous work on renal fibroblasts derived from rodents, and indicates that the effects of TGF-β1 on fibroblasts are mediated by the TGF-β1RI(ALK5)/TGF-β1RII receptor complex in cats as in other species [54].

There were several limitations to the current study, most significantly, the small number of cats included. It is possible that the conclusions drawn regarding cell marker expression were skewed due to the small number of isolations, and further investigation utilising a larger population of cats is required to support the findings of this study. There was particular difficulty in obtaining kidney tissue from cats with normal kidney function, and deriving FCF cells from this tissue. Therefore it was not possible to obtain enough FCF to perform TGF-β1 stimulation experiments on this cell type. It is possible that fibroblasts from diseased kidneys have a heightened sensitivity to TGF-β1, and enhanced coupling to pathways regulating gene expression, but a definitive demonstration of this is currently not possible. Further work to isolate larger numbers of FCF would enable direct comparison of data between FCF and CKD-FCF, and potentially provide new insights into the pathology of feline CKD.

A further limitation was that the CKD-FCF were obtained from cats of different ages and, furthermore, with differing degrees of renal pathology. This diverse origin is likely responsible for the variability in the behaviour of cells from different animals. These factors were largely unavoidable due to the isolation of these cells from pet cats euthanized for welfare reasons (with owner informed consent). Furthermore recombinant human TGF-β1 was used in experiments, as feline TGF-β1 is not commercially available. TGF-β1 is a well-conserved gene, with 95.1% homology between the human and feline sequence, which is also true of the TGF-β1RI gene (92.7% homology) and, to a lesser extent, the TGF-β1RII gene (83.8%) [55], but receptor-ligand kinetics may have been affected by this different species of origin. Furthermore, only mRNA expression after incubation with TGF-β1 was examined, which may not have accurately represented protein expression. Further work to confirm increased protein expression of the target genes should also be performed in order to confirm these results. Finally, the in vitro cell culture environment is significantly different from the three dimensional kidney architecture, within which cells reside in complex structures. Therefore, these results obtained in vitro do need to be translated with caution into the whole animal.

## Conclusions

In conclusion, we have reported a simple methodology for the isolation of FCF/CKD-FCF from cadaverous kidney tissue. The cultures described represent an in vitro model for the investigation of factors and processes relevant to fibroblast-myofibroblast differentiation, and by extension, the initiation and progression of renal fibrosis in vivo. FCF and CKD-FCF appeared phenotypically similar, although expression of VCAM-1 on CKD-FCF suggested these cells were in an “activated” state. In addition, TGF-β1 was shown to induce dose dependent alterations in morphology and gene expression in CKD-FCF suggestive of myofibroblast differentiation. These effects were inhibited by the TGF-β1RI antagonist SB431542, suggesting they are mediated via the TGF-β1RI (ALK5)/TGF-β1RII receptor complex. These findings suggest that TGF-β1 may be involved in fibroblast-myofibroblast transition in feline chronic kidney disease as in other species.

## Abbreviations

CKD: Chronic kidney disease
CKD-FCF: Feline cortical fibroblasts from cats with CKD
CTGF: Connective tissue growth factor
DMEM/F12: Dulbecco’s modified Eagle medium: Nutrient mixture F12
DPBS: Dulbecco’s phosphate buffered saline
ECM: Extra-cellular matrix
FBS: Foetal bovine serum
FCF: Feline cortical fibroblasts from cats with normal renal function
TBS: Tris-buffered saline
TGF-β1: Transforming growth factor β1
TGF-β1RII: TGF-β1 type II receptor
VCAM-1: Vascular cell adhesion molecule 1
vWF: Von Willebrand factor
α-SMA: Alpha smooth muscle actin
